# Dynamic Taping Improves Landing Biomechanics in Young Volleyball Athletes

**DOI:** 10.3390/ijerph192013716

**Published:** 2022-10-21

**Authors:** Chih-Kuan Wu, Yin-Chou Lin, Chi-Ping Lai, Hsin-Ping Wang, Tsung-Hsun Hsieh

**Affiliations:** 1 Department of Physical Medicine and Rehabilitation, Chang Gung Memorial Hospital, Linkou, Taoyuan 33305, Taiwan; 2 Center of Comprehensive Sports Medicine, Chang Gung Memorial Hospital, Linkou, Taoyuan 33305, Taiwan; 3 School of Physical Therapy and Graduate Institute of Rehabilitation Science, Chang Gung University, Taoyuan 33302, Taiwan; 4 Neuroscience Research Center, Chang Gung Memorial Hospital, Linkou, Taoyuan 33305, Taiwan; 5 Healthy Aging Research Center, Chang Gung University, Taoyuan 33305, Taiwan

**Keywords:** dynamic taping, landing error scoring system (LESS), laxity, athletes

## Abstract

Poor landing biomechanics such as hip adduction, internal rotation, and knee valgus have been recognized as modifiable risk factors of anterior cruciate ligament (ACL) injury. Dynamic taping is a newly developed technique with better elasticity and extensibility, which could change the landing biomechanics. The purpose of this study was to identify whether dynamic taping could improve lower limb biomechanics in athletes. Forty-two high school volleyball athletes (21 males and 21 females) participated in the study. Biomechanical properties, including the landing error scoring system (LESS) and anterior–posterior knee laxity, were evaluated before and after the application of dynamic tape while athletes performed the jump-landing task. As a result, we found that dynamic tape significantly reduced the faulty landing strategy by an average of 0.64 errors in all volleyball athletes. The effect induced by dynamic tape was more prominent in female athletes and high-risk athletes (1.1 errors). Furthermore, the application of dynamic tape improved anterior–posterior knee laxity, especially in female athletes (*p* < 0.001). In conclusion, we found that dynamic tape provided a short-term, passive, and clinically significant means to normalize inadequate biomechanics during landing in athlete groups, which could have a protective effect and further alleviate the risk of ACL injury.

## 1. Introduction

An anterior cruciate ligament (ACL) injury is one of the most serious sports injuries. In addition to the significant financial burden, the subsequent rehabilitation program, functional limitation, and decreased performance in sports causes more stress for competitive athletes. Approximately 70% of ACL injuries occur in noncontact mechanisms, such as cutting, stopping suddenly, and landing [1,2]. The incidence is highest in basketball, handball, soccer, and volleyball. Among the noncontact mechanisms, the number of landing-related injuries is significantly higher than cutting and stopping, especially in volleyball athletes in an Asian study [2].

According to previous research, ACL injury is multifactorial and includes anatomic risk factors [3,4,5], hormonal risk factors [6,7,8], biomechanical risk factors [9,10,11,12,13], and unanticipated factors, such as fatigue, misjudgment, or falls [9]. Among biomechanical risk factors, increased anterior tibial shear force [10], decreased knee flexion while landing [11], increased knee valgus [12], increased knee and hip joint internal rotation [13], and increased hip adduction [14] were found and have become the main research issues. Therefore, from the perspective of preventing ACL injuries, biomechanical risk factors are modifiable and could be adjusted by suitable exercise or intervention, such as taping. Moreover, knee laxity is an important research target related to anatomic and biomechanical risk factors. As reported by Sadra, high-risk landing biomechanics are associated with exercise-related knee laxity [15]. In females, increased anterior–posterior knee laxity is related to greater knee internal rotation motion during landing, whereas anterior–posterior knee laxity is related to knee stiffness in males. A previous study also showed that generalized joint laxity was a predictor of ACL injuries [5].

According to previous studies, the landing error scoring system (LESS) is a clinical assessment tool to perform field evaluation with good sensitivity (86%) and specificity (64%) [10,16]. The one-season risk of ACL injury in athletes with LESS scores of 5 or more is 1.37% [16]. The risk in athletes with a LESS score less than 5 is 0.13% [16]. Current evidence shows good to excellent intrarater and interrater reliabilities and moderate to excellent agreement between key knee injury risk factors and ACL injury [17]. The scoring criteria identify subjects with faulty landing biomechanics or high-risk movement patterns, such as increased knee valgus, hip internal rotation and adduction during initial contact, and maximal knee flexion. Moreover, the asymmetric landing of feet and side bending of the trunk in the frontal view and decreased sagittal plain joint flexion of the trunk, hip, and knee could be identified [18,19]. A higher LESS score indicates a more faulty jump-landing technique, which represents a higher risk of ACL injury. Therefore, LESS is a predictor of ACL injury [16].

There are many kinds of tape commonly used by athletes, such as rigid athletic tapes, kinesiology tapes, and leukotapes. Compared to Kinesio tape and rigid tape, dynamic taping is a newly developed technique with better elasticity and multidirectional extensibility, no rigid endpoint, and stronger resistance and recoil [20], providing somatosensory input and enhancing proprioception in the previous research [21]. However, clinical research is extremely scarce. One abstract report revealed that dynamic taping with the spiral technique applied to athletes could decrease the frontal plane projection angle during the single-leg squat [22]. Another blinded randomized controlled trial showed that dynamic taping with the spiral technique applied to patients with greater trochanteric pain syndrome could reduce the hip adduction moment and adduction angle while walking [23]. Therefore, dynamic taping appears to have a biomechanical effect on lower limb movement patterns.

To the best of our knowledge, no study has examined the effects of dynamic taping on landing biomechanics. The purpose of this study was to determine the relationship between dynamic taping intervention and changes in the LESS score and knee laxity. It is hypothesized that dynamic taping could improve lower limb biomechanics and knee laxity in athletes. The knowledge obtained in the current study may provide a new approach to modifying lower limb biomechanics and decreasing the ACL injury rate.

## 2. Materials and Methods

### 2.1. Participants

Forty-two high school volleyball athletes participated in this study (Table 1). These athletes engaged in volleyball training five times per week. None of the participants had suffered from ACL injuries or received ACL reconstruction before. Previous knee injuries were not exclusion criteria; however, all participants at the initial test were free from an acute medical condition that would prohibit volleyball training and competition, and there was no reported pain or discomfort during the experimental procedures. Ethical approval for this study was received from Chang Gung Medical Foundation Institutional Review Board, and all participants and their legal guardians provided written informed assent and consent (No: 201901447B0).

### 2.2. Procedures

**Figure 1:**
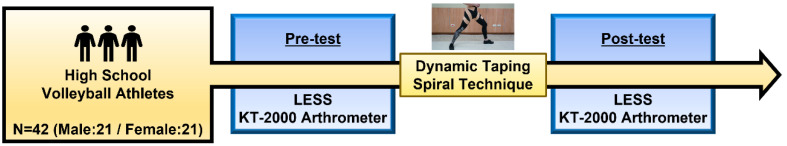
Experiment flowchart. Forty-two participants completed LESS and knee laxity examinations with a KT2000 arthrometer and then received dynamic taping. After taping, measurements of LESS and knee laxity were repeated immediately.

All athletes completed a baseline questionnaire that addressed sport-related injury history and demographic data, such as body height and weight. The entire examination was performed before the regular training program. Participants completed LESS and knee laxity examinations with a KT2000 arthrometer and then received dynamic taping. After taping, measurements of LESS and knee laxity were repeated immediately (Figure 1). All procedures, including jump tasks and dynamic taping, were performed in the high school health center.

### 2.3. Landing Error Scoring System

The LESS is a screening field test to evaluate the individual landing technique by 17 items [24]. Participants wore comfortable shoes during all test procedures. Before the task, all participants had as many practice trials as needed, and no feedback or teaching on their landing strategy was given during the task. For the task, they jumped from a 30-cm-high box to a designated landing area, which was a distance equivalent to 50% of their body height, and immediately jumped vertically as high as possible. All subjects completed three trials of the jump-landing task before and after the dynamic taping intervention.

We recorded the entire jump-landing test from frontal and lateral views using two standard Handycams (Sony DCR-CX900 hard disk drive camera, Tokyo, Japan). After collecting the data, the LESS was rated independently by an experienced rater, with the dominant leg as the primary target. The experienced rater is a physical medicine and rehabilitation physician with more than 10 years of experience in functional movement evaluation and sports medicine. The intrarater reliability of the rater was high ((ICC)_2,1_ = 0.916). To rate the LESS, we reviewed frontal and sagittal views frame by frame. These frames included low limb position and posture at initial contact of the foot (Items 1–6), foot position while the entire foot was on the ground (Items 7 and 8), maximal knee flexion (Items 9 and 10), and the symmetry of landing (Item 11). Then, trunk and low limb joint displacement were assessed from initial contact to maximal knee flexion (Items 12–15). Finally, joint displacement and overall impression of the entire landing task were evaluated (Items 16 and 17). Participants who scored an error in 2 of 3 trials received an error in the LESS items.

### 2.4. Knee Laxity

A KT-2000 arthrometer (Medmetric, San Diego, CA) was applied to measure anterior–posterior knee laxity. Participants laid down on the examination table in the supine position and knee flexion 30 ± 5 degree and 15–25 degree external rotation over thigh support [25]. The leg was secured in place with circumferential Velcro straps. The research assistant applied a forward force three times and recorded the maximal value in both legs. There were three examinations to measure anterior–posterior knee laxity: (1) anterior displacement with a 20-pound force (89 N), (2) compliance index: anterior displacement between a 15- and 20-pound force, and (3) anterior displacement with a 30-pound force (134 N). Similar to LESS, the dominant leg was the primary target. One well-trained examiner performed all the knee laxity tests. To understand the consistency of measures between time points, we also calculated the intrarater reliability for the knee laxity test using a 2-way mixed-effects model [26]. Briefly, the single rater independently assessed the laxity of the knee joint under 20-pound forces three times per session and recorded the maximal value in both legs. Testing occurred in two sessions, seven days apart. The high ICCs values for knee laxity value under 20-pound force were obtained in the left and right leg (ICC = 0.924 and 0.884, respectively), suggesting that the protocols of such measurement were reliable.

### 2.5. Dynamic Taping

**Figure 2:**
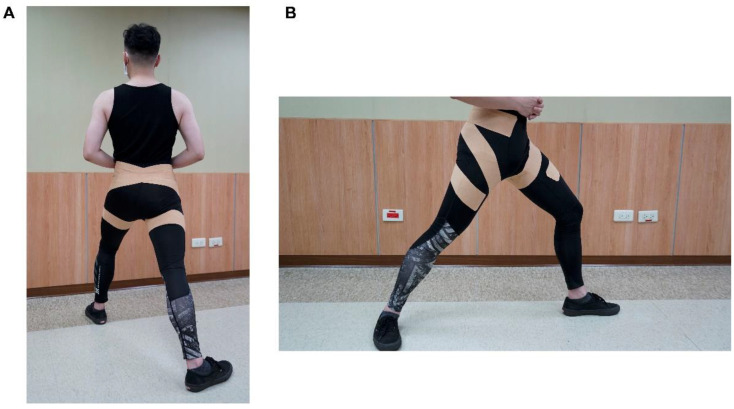
Demonstration of the dynamic tape applied on the bilateral hip. (**A**) Posterior view, (**B**) side view. The dynamic tape started from the vastus medialis obliquus and wrapped around the thigh in a superolateral direction to the posterior thigh, went to the proximal medial thigh, and then wrapped the thigh below the anterior superior iliac crest in a superolateral direction. After that, the dynamic tape crossed the low back to the contra-lateral lower quarter abdomen. The dynamic tape was applied on both hip joints.

The dynamic tape was used by the same experienced athletic trainer and directly applied to the skin. A spiral double-layer of 7.5 cm (Powerband) dynamic tape was applied on the bilateral hip, and the hip was placed in 40° abduction, 20° extension, and full available external rotation, aiming to resist hip adduction, flexion, and internal rotation. The dynamic tape was applied bilaterally for LESS items to evaluate bilaterally, such as stance width, lateral trunk flexion, and overall impression. The powerband was created by applying additional dynamic tape length in parallel [20] (Figure 2a,b).

### 2.6. Statistical Analyses

A Shapiro–Wilks test was conducted to assess the normality of measurements in LESS and laxity. We used the paired t-test to compare the effects of dynamic taping on the LESS total score and each specific scoring item. In addition, paired t-test was used to compare knee laxity before and after dynamic tape application. All statistical analyses were performed in IBM SPSS (Version 24.0; IBM Corporation, Amrmonk, NY, USA). Cohen’s d effect sizes were also calculated with G*Power to aid in the interpretation of the results. The magnitude of the effect size was interpreted using thresholds as suggested by Cohen: 0.0 to 0.19—trivial; 0.20 to 0.49—small; 0.50 to 0.79—moderate; >0.80—large [27]. The level of significance for all statistical tests was set as 0.05.

## 3. Results

**Figure 3:**
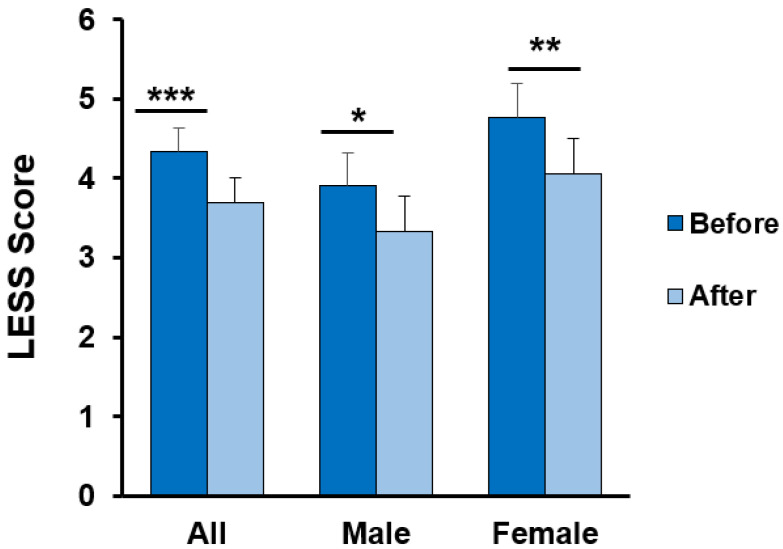
Comparison of the LESS score before and after dynamic taping (mean ± SE). * *p* < 0.05, ** *p* < 0.01, and *** *p* < 0.001; each indicates a significant difference before and after taping.

It has been found that all the current data were normally distributed after running the Shapiro–Wilk test. Therefore, the paired t-test was applied to compare the effects of dynamic taping. The LESS score of all athletes decreased from 4.33 ± 1.98 to 3.69 ± 2.07 (t = 3.95, *p* < 0.001, effect size = 0.75) after dynamic tape application. The difference was present in both sexes (male: from 3.90 ± 1.95 to 3.33 ± 2.06, t = 2.17, *p* = 0.04, effect size = 0.74, female: from 4.76 ± 1.97 to 4.05 ± 2.06, t = 3.63, *p* = 0.002, effect size = 1.07) (Table 2, Figure 3).

For the specific LESS items, the frequency of a faulty landing strategy in all athletes decreased in joint displacement and overall impression (Items 16 and 17) after applying the dynamic tape. Items 1–15 did not reach statistical significance and are shown in Table 3. The LESS score in the high-risk group showed greater improvement compared with the low-risk group (1.1 points in the group with LESS ≥ 6 group and 0.5 points in the group with LESS ≤ 5) (Table 4).

**Figure 4:**
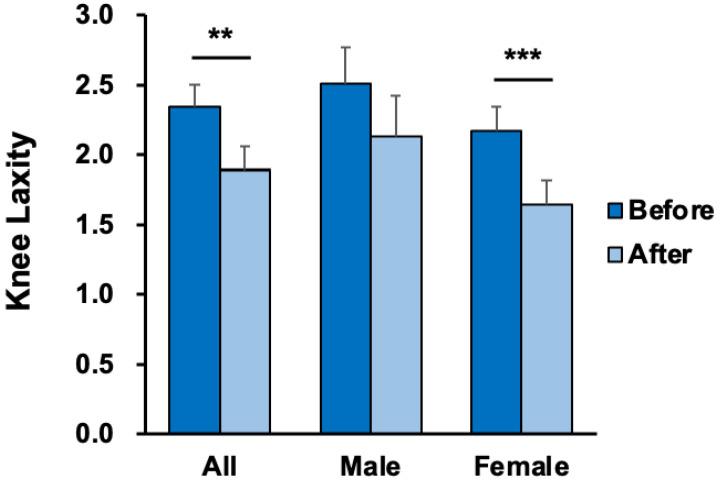
Comparison of anterior–posterior knee laxity of the dominant leg before and after dynamic taping (mean ± SE). ** *p* < 0.01, and *** *p* < 0.001; each indicates a significant difference before and after taping.

**Figure 5:**
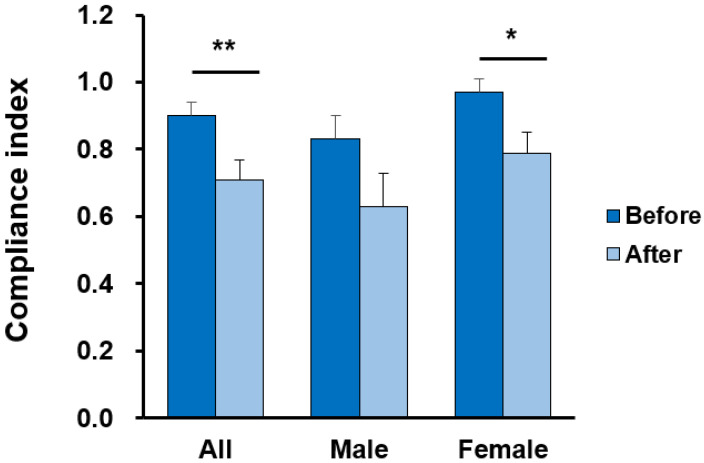
Comparison of compliance index of the dominant leg before and after dynamic taping (mean ± SE). * *p* < 0.05 and ** *p* < 0.01; each indicates a significant difference before and after taping.

The anterior–posterior knee laxity of the dominant leg under a 20-pound force decreased from 2.34 ± 1.01 to 1.89 ± 1.12 mm (t = 3.31, *p* = 0.002, effect size = 0.64) in all athletes and from 2.17 ± 0.76 to 1.65 ± 0.78 mm (t = 4.90, *p* < 0.001, effect size = 1.29) in female athletes after applying dynamic tape. The compliance index of the dominant leg also decreased from 0.90 ± 0.27 to 0.71 ± 0.38 mm (t = 2.93, *p* = 0.006, effect size = 0.59) in all athletes and from 0.97 ± 0.21 to 0.79 ± 0.29 mm (t = 2.45, *p* = 0.02, effect size = 0.72) in female athletes. The anterior–posterior knee laxity of the dominant leg under a 30-pound force decreased from 3.13 ± 1.24 to 2.63 ± 1.23 mm (t = 3.16, *p* = 0.003, effect size = 0.62) in all athletes and from 2.93 ± 1.01 to 2.30 ± 0.85 mm (t = 3.94, *p* < 0.001, effect size = 1.07) in female athletes. However, neither result reached statistical significance in male athletes (Table 5, Figure 4 and Figure 5).

## 4. Discussion

The current study is the first to examine the effects of dynamic taping on the LESS. We found that applying dynamic tape decreased the faulty landing strategy by an average of 0.64 errors (14.8% lower) in all volleyball athletes, and the effect of dynamic taping was more prominent in the female athletes (effect size: 1.07). Moreover, previous research showed that the minimal clinical important difference (MCID) of LESS is around 1.16 [28]. In the current study, the effect of dynamic taping in the high-risk group (LESS ≥ 6) also reached close to the MCID. According to our results, the application of dynamic tape to the bilateral hip joint also decreased anterior–posterior knee laxity in three examinations of knee laxity, especially in female athletes with a larger effect size. These findings support our hypotheses that dynamic taping could change the LESS score and knee laxity in volleyball athletes.

Previous studies have demonstrated the change in LESS in patients with ACL reconstruction [29,30]. The LESS score was higher in ACL reconstruction (6.7) compared to controls (5.6) and was more likely to have an error for lateral trunk flexion during landing [30]. There are also extrinsic factors that could modify landing biomechanics. More hip and knee extension and more knee valgus and anterior tibial shear force were observed in the fatigue status [31,32]. Previous studies reported that after a short-term functional fatigue protocol, the LESS score was higher in both healthy subjects and patients with ACL reconstruction. In these findings, LESS presents as a clinical tool to quantify the change in biomechanics in ACL-reconstructed and healthy athletes [31,33,34].

As a screening tool, the total score of the LESS was higher in those who sustained an ACL injury. In the clinical setting, receiver operator characteristic analysis reveals that the cutoff point of the LESS score of 5 has an ideal screening ability: 86% sensitivity and 64% specificity. Padua and colleagues showed that the most predictive items in the LESS for noncontact ACL injuries are trunk-flexion displacement, joint displacement, foot position, and external rotation [16]. In our study, the faulty movement in joint displacement and the overall impression were reduced significantly after applying the dynamic tape (Table 3). Moreover, knee valgus has been suggested as a prospective biomechanical risk factor for ACL injuries [12]. Our observation showed that the faulty movement of the knee position at the initial contact and landing phases improved close to statistical significance (*p* = 0.06 and 0.10, respectively). The dynamic tape was applied on the bilateral hip in full external rotation to resist hip adduction and internal rotation during landing. Previous studies also demonstrated that greater hip external rotator strength promotes better dynamic control of the femur and pelvis during the landing phase of unanticipated tasks [35] or single-leg landings [14]. Our study’s results support the same findings.

Knee laxity has been reported as a biomechanical risk factor for ACL injuries during landing [5,6,15,36]. Our results revealed that applying dynamic tape on the hip joint also improves anterior–posterior knee laxity. To our knowledge, no study has demonstrated the relationship between femoral taping and knee laxity. There are three possible explanations: first, previous research revealed that Kinesio taping applied to the hip joint with maximal external rotation would shift the patella distally and posteriorly [37]. This kinematic change in the patella would increase the contact area between the patella and femur, which may affect the measurement of knee laxity. Second, the application of dynamic tape on a hip joint could reduce hip adduction and internal rotation, which would ameliorate the compression of the iliotibial band [23]. The iliotibial band plays a role in anterior–posterior and lateral laxity [38]. Third, dynamic tape presented good multidirectional elasticity, providing somatosensory input and enhancing proprioception in previous research [21]. Furthermore, proprioception is correlated with knee laxity [39].

With regard to the anterior displacement under testing with 20- or 30-pound forces, it is found that the displacement value under the 30-pound force test is smaller than the values reported by earlier studies [11,40,41,42,43] while the mean value in the anterior displacement with a 20-pound force is close to previous studies [41,42,44]. The smaller displacement value under the 30-pound force test could have resulted from the thigh muscles, which were not completely relaxed or under co-contraction during examination [45,46]. Although the subjects were asked to relax their leg muscles throughout testing, advanced strategies were considered to be applied to avoid these potential biases during the laxity test. For example, if subjects had difficulty accomplishing the relaxation of their thigh muscles, the examiner could help them achieve the needed relaxation by performing gentle anterior–posterior oscillation of their lower legs [10]. In addition, to further confirm the relaxation level of the thigh muscles before and during the examination, the electromyography (EMG) measurement could be applied for biofeedback to enhance muscle relaxation [47,48].

Female athletes have a higher risk of ACL injuries than male athletes in the same sports [49,50,51]. The sex disparity in the ACL injury rate is obvious in adolescence and diminishes gradually in adulthood [52]. In different sports, gender differences vary. The female-to-male ratio of ACL injury in volleyball athletes is 7.2 (6.2–8.3, 95% CI), which is much higher than the 3.3 (6.2–8.3, 95% CI) of average high school athletes [51]. In our study, the LESS score was higher in the female group, which is compatible with previous research [10,16,53]. However, the effects of dynamic taping were effective in all athletes.

Several kinds of exercise training have been advocated to prevent ACL injury, and the most well-known injury prevention program is the FIFA 11+ program for soccer athletes [54]. However, a systematic review and meta-analysis demonstrated that ACL and knee injury prevention programs decrease lower limb injuries, but no significant change was found in the ACL injury rate. The most challenging part of the program may be the compliance of the coach and athletes. A previous study showed that the compliance of soccer coaches to implement ACL prevention programs was 19.8% [55]. Therefore, finding high-risk athletes and applying dynamic taping to normalize the inadequate biomechanics during landing could be one of the supportive approaches for athletes.

There are limitations to this study. First, this was not a randomized controlled trial, and we could not rule out the placebo effect or proprioceptive factor from the mechanical effects of dynamic tape. Second, some differences before and after intervention did not reach statistical significance, which may be related to the small sample size. Third, our subjects were healthy volleyball athletes and categorized as low risk with an average LESS score. Evaluating the effect in the impaired population such as patients with anterior cruciate ligament injuries could be a future target. Fourth, in the current study design, the rater could be biased by the presence of the tape while rating the LESS score. Comparison groups with sham-taping or covering with clothing could decrease the research bias in future studies. Thus, the quality of evidence is limited.

## 5. Conclusions

The results of this study support the hypothesis that the application of dynamic tape to the hip joint improves the low limb biomechanics during landing and knee laxity. The effects were more prominent in female athletes and high-risk athletes. For the uncertain compliance of exercise intervention, the passive application of dynamic tape could be a useful tool. The practical implication for athletes, coaches, and athletic trainers is that dynamic tape could provide a short-term, passive, and supportive tool to correct faulty biomechanics in athlete groups.

## Figures and Tables

**Table 1:** Demographic data of participants.

	Male (*n* = 21)	Female (*n* = 21)	*p-*Value
Age (Year)	16.48 ± 0.81	16.14 ± 0.96	0.23
Body Height (cm)	178.00 ± 5.52	164.43 ± 5.64	<0.001 *
Body Weight (Kg)	70.87 ± 5.96	59.87 ± 7.05	<0.001 *
BMI (Kg/cm^2^)	22.31 ± 1.41	22.09 ± 2.35	0.71
Dominant Leg (Right/Left)	19/2	19/2	

BMI = body mass index. * Significant difference (*p* < 0.05) between male and female athletes. All data are reported as mean ± SD.

**Table 2:** The LESS scores of participants before and after dynamic taping.

	Before	After	*p-*Value	*t* Value	Effect Size
All athletes (*n* = 42)	4.33 ± 1.98	3.69 ± 2.07	<0.001 ***	3.95	0.75
Male athletes (*n* = 21)	3.90 ± 1.95	3.33 ± 2.06	0.04 *	2.17	0.74
Female athletes (*n* = 21)	4.76 ± 1.97	4.05 ± 2.06	0.002 **	3.63	1.07

Significant difference (* *p* < 0.05, ** *p* < 0.01, and *** *p* < 0.001) before and after taping. All data are reported as mean ± SD.

**Table 3:** The difference before and after applying dynamic tape in individual items.

Landing Error Scoring System		Before (*n* = 42)	After (*n* = 42)	*p-*Value
#1	Knee flexion: initial contact	0.50 ± 0.51	0.45 ± 0.50	0.49
#2	Hip flexion: initial contact	0.02 ± 0.15	0.00 ± 0.00	1.00
#3	Trunk flexion: initial contact	0.31 ± 0.47	0.26 ± 0.45	0.32
#4	Ankle plantar flexion: initial contact	0.17 ± 0.38	0.12 ± 0.33	0.32
#5	Medial knee position: initial contact	0.43 ± 0.50	0.33 ± 0.48	0.10
#6	Lateral trunk flexion: initial contact	0.00 ± 0.00	0.00 ± 0.00	N/A
#7	Stance width: wide	0.14 ± 0.35	0.07 ± 0.26	0.18
#8	Stance width: narrow	0.19 ± 0.40	0.19 ± 0.40	1.00
#9	Foot position: external rotation	0.00 ± 0.00	0.00 ± 0.00	N/A
#10	Foot position: internal rotation	0.24 ± 0.43	0.19 ± 0.40	0.18
#11	Symmetric initial foot contact: initial contact	0.07 ± 0.26	0.10 ± 0.30	0.66
#12	Knee-flexion displacement	0.00 ± 0.00	0.00 ± 0.00	N/A
#13	Hip-flexion displacement	0.00 ± 0.00	0.00 ± 0.00	N/A
#14	Trunk-flexion displacement	0.24 ± 0.43	0.17 ± 0.38	0.32
#15	Medial-knee displacement	0.76 ± 0.43	0.64 ± 0.48	0.06
#16	Joint displacement	0.57 ± 0.50	0.48 ± 0.51	0.04 *
#17	Overall impression	0.76 ± 0.43	0.67 ± 0.48	0.04 *

N/A = not applicable. * Significant difference (*p* < 0.05) before and after taping. All data are reported as mean ± SD.

**Table 4:** Subgroup analysis of the high- and low-risk groups before and after taping.

	Before	After	*p*-Value	*t* Value	Effect Size
LESS ≥ 6 (*n* = 10)	7.00 ± 0.94	5.90 ± 1.85	0.02 *	2.70	1.14
LESS ≤ 5 (*n* = 32)	3.50 ± 1.39	3.00 ± 1.61	0.002 **	2.98	0.68

Significant difference (* *p* < 0.05, ** *p* < 0.01) before and after taping. All data are reported as mean ± SD.

**Table 5:** The anterior–posterior knee laxity and compliance index of the dominant leg before and after dynamic taping.

		Before (mm)	After (mm)	*p*-Value	*t* Value	Effect Size
Knee Laxity under 20-pound force	All athletes (*n* = 42)	2.34 ± 1.01	1.89 ± 1.12	0.002 *	3.31	0.64
	Male athletes (*n* = 21)	2.51 ± 1.20	2.13 ± 1.35	0.15	1.50	0.51
	Female Athletes (*n* = 21)	2.17 ± 0.76	1.65 ± 0.78	<0.001 ***	4.90	1.29
Compliance Index	All athletes (*n* = 42)	0.90 ± 0.27	0.71 ± 0.38	0.006 **	2.93	0.59
	Male athletes (*n* = 21)	0.83 ± 0.31	0.63 ± 0.45	0.08	1.83	0.59
	Female Athletes (*n* = 21)	0.97 ± 0.21	0.79 ± 0.29	0.02 *	2.45	0.72
Knee Laxity under 30-pound force	All athletes (*n* = 42)	3.13 ± 1.24	2.63 ± 1.23	0.003 *	3.16	0.62
	Male athletes (*n* = 21)	3.33 ± 1.42	2.97 ± 1.46	0.20	1.34	0.48
	Female Athletes (*n* = 21)	2.93 ± 1.01	2.30 ± 0.85	<0.001 ***	3.94	1.07

Significant difference (* *p* < 0.05, ** *p* < 0.01, *** *p* < 0.001) before and after taping. All data are reported as mean ± SD.

## Data Availability

Not applicable.
